# Novel multimodel ensemble approach to evaluate the sole effect of elevated CO_2_ on winter wheat productivity

**DOI:** 10.1038/s41598-019-44251-x

**Published:** 2019-05-24

**Authors:** Mukhtar Ahmed, Claudio O. Stöckle, Roger Nelson, Stewart Higgins, Shakeel Ahmad, Muhammad Ali Raza

**Affiliations:** 1 Department of Agronomy, PMAS Arid Agriculture University, Rawalpindi, 46300 Pakistan; 2 Department of Biological Systems Engineering, Washington State University, Pullman, WA 99164-6120 USA; 3 Department of Northern Agricultural Sciences, Swedish University of Agricultural Sciences, Umeå, 90183 Sweden; 4 Department of Agronomy, Faculty of Agricultural Sciences and Technology, Bahauddin Zakariya University, Multan, 60800 Pakistan; 5 College of Agronomy, Sichuan Agricultural University, Chengdu, 611130 PR China

**Keywords:** Plant ecology, Agroecology

## Abstract

Elevated carbon-dioxide concentration [eCO_2_] is a key climate change factor affecting plant growth and yield. Conventionally, crop modeling work has evaluated the effect of climatic parameters on crop growth, without considering CO_2_. It is conjectured that a novel multimodal ensemble approach may improve the accuracy of modelled responses to eCO_2_. To demonstrate the applicability of a multimodel ensemble of crop models to simulation of eCO_2_, APSIM, CropSyst, DSSAT, EPIC and STICS were calibrated to observed data for crop phenology, biomass and yield. Significant variability in simulated biomass production was shown among the models particularly at dryland sites (44%) compared to the irrigated site (22%). Increased yield was observed for all models with the highest average yield at dryland site by EPIC (49%) and lowest under irrigated conditions (17%) by APSIM and CropSyst. For the ensemble, maximum yield was 45% for the dryland site and a minimum 22% at the irrigated site. We concluded from our study that process-based crop models have variability in the simulation of crop response to [eCO_2_] with greater difference under water-stressed conditions. We recommend the use of ensembles to improve accuracy in modeled responses to [eCO_2_].

## Introduction

Climate change and food security are two interlinked challenges faced by human beings in the 21^st^ century^1^. World agriculture is under the influence of climate change and it is facing daunting challenges to meet the food, fuel and fiber demands. Climate trends across the globe reveal that crop production might be under stress in spite of technological advances. Concentration of [CO_2_] has increased from 280 ppm before the industrial revolution to 411.91 ppm now^2^. Projections of [CO_2_] at 2100 range from 500–1000 ppm^3^. The [CO_2_] may rise to 1000 ppm by 2100 with a 2–4 °C increase in temperature along with variable precipitation and more frequent, intense and longer extreme events^4^. Houghton *et al*.^5^ reported that [CO_2_] increased by 35% due to fossil fuel burning and land use change from 1990 to 2010. The BERN climate change model projected that [CO_2_] will change from 390 ppm to 700–1000 ppm with climate change at the end of century^6^. Trenberth and Jones^7^ projected surface temperature increase of 0.74 ± 0.18 °C due to elevated concentration [eCO_2_]. Global warming due to [eCO_2_] could change earth’s surface temperature from 0.4–2.6 °C in the 2046–2065 window and from 0.3–4.8 °C between 2081 and 2100 in comparison to the 1986–2005 baseline^8^. The rise in [CO_2_] will likely result in increased photosynthesis, reduced stomatal conductance and transpiration, and ultimately higher water- and light-use efficiency in plants^9–12^.

Carbon dioxide is an important substrate of photosynthesis and its elevated concentration results in metabolic changes in crops directly through photosynthesis (A) and stomatal conductance (g_s_)^13^. Crops having the C_3_ photosynthetic pathway, currently have suboptimal [CO_2_], but under [eCO_2_], photosynthesis might be stimulated. C_3_ crops have the potential to capitalize on [eCO_2_] by increasing photosynthetic rates and, thus, provide better growth and yield^14^. Elevated CO_2_ (CO_2_ fertilization effect) will be beneficial for Ribulose 1,5-bisphosphate carboxylase/oxygenase (Rubisco) and may inhibit photorespiration and increase photosynthesis. Ainsworth and Rogers^15^ concluded that [eCO_2_] stimulated light-saturated photosynthesis by 31% and reduced stomatal conductance by 22% in free-air CO_2_ enrichment (FACE) experiments. Meanwhile, Kruijt *et al*.^16^ concluded that at [eCO_2_], stomatal activity is reduced. This change in stomatal activity resulted in a 50% increase in water use efficiency. Varga *et al*.^10^ reported increased water use efficiency in winter wheat under stress conditions due to [eCO_2_] (700–1000 ppm). Photosynthesis increases with [eCO_2_] following a Michaelis-Menten curve. The Michaelis–Menten constant (*K*_*m*_) could be used to quantify the [eCO_2_] effects under different temperatures^17^. Almost 23% of the carbon fixed by photosynthesis is lost due to photorespiration and if it is stopped completely, the carboxylation reaction could increase to 53%. It is suggested that with the future rise in [CO_2_] Rubisco will have higher *Km* (6.3 to 15 µM) resulting in higher photosynthetic rate and efficiency^15^. Tausz *et al*.^14^ concluded that photosynthetic acclimation or photosynthetic downregulation could be inhibited by [eCO_2_]. Similarly, [eCO_2_] resulted in a decrease in evapotranspiration (ET)^18^. Transpiration efficiency could be improved by increased net photosynthesis and reduced stomatal conductance^19^. Radiation use efficiency (RUE) can be defined as biomass produced per unit of light energy used by crops. Different process-based crop models use RUE-based functions to moderate the effect of [eCO_2_] on biomass accumulation. Yin and Struik^20^ proposed a new framework to quantify the conversion efficiency of incident solar radiation into phytoenergy by annual crops. They indicated that for C_3_ crops the overall efficiency of converting incident solar radiation into phytoenergy was 2.2% (RUE = 1.22 g MJ^−1^) under 400 μmol mol^−1^ [CO_2_] which could be increased to 3.6% (RUE = 1.75 g MJ^−1^).

Different approaches have been used to study the effect of [eCO_2_] on plants growth, development and yield. These include FACE experiments^9,12,21^, open top chamber (OTC)^22–24^, temperature gradient tunnel (TGT)^25^ and crop modeling. The FACE approach is considered more appropriate compared to other experimental approaches as it can provide data that better resemble field conditions. In general, C_3_ cereal crop response to [eCO_2_] under water stress is comparatively higher (22%) than under irrigated conditions (16%)^26^. Similarly, increased photosynthesis (10–45%) in C_3_ crops with increased canopy temperature, yield, biomass and water use efficiency and decreased stomatal conductance and evapotranspiration have been reported under FACE experiments^9^. Wheat, the main C_3_ cereal crop, showed reduced stomatal conductance and evapotranspiration with increased photosynthesis and canopy temperature under [eCO_2_]. This resulted in higher biomass and yield in wheat even under water stress conditions. Hocking and Meyer^27^ reported doubled drymatter production in wheat under [eCO_2_] treatments compared to control. Meanwhile, higher water use efficiency (19–23%) under the high N treatment was reported in wheat under FACE^28^. FACE experiments from Australia and China reported a 21–23% increase in biomass and 24.8% increase in wheat grain yield under [eCO_2_]^29^.

Crop simulation models often used to study crop behavior under changing climate. Many researchers have used crop modeling under different climatic scenarios^16,26,30–42^. The Agricultural Model Intercomparison and Improvement Project (AgMIP) studied the impact of climate change on agricultural production and food security using process-based crop models^43^. Crop model comparison under the AgMIP framework revealed that uncertainties in wheat yield simulation increased with increased temperature-by-CO_2_ interactions^44^. Similarly, Asseng *et al*.^39^ tested 30 different wheat crop models in response to elevated temperature and predicted that most of the models simulated yield well under baseline conditions, but with increasing spread at the higher future temperatures. Furthermore, Rosenzweig *et al*.^45^ found strong negative effects of climate change particularly at higher temperature. However, they recommended further research to minimize uncertainties related to the representation of carbon dioxide, nitrogen, and high temperature. Most of the earlier crop modeling work was focused more on studying and quantifying the impact of temperature on crop growth, development and yield^46–50^. They, in general, found a reduction in grain yield with some level of uncertainty under higher temperature. Similarly, earlier modeling studies focused on the combined effect of climatic parameters i.e. [eCO_2_], temperature, nitrogen and drought^9,24,34,51,52^. Some of the earlier work studied the interaction of increased temperature and CO_2_^53–55^.

The interaction between increasing temperature and [eCO_2_] is difficult to isolate, and the interpretation of projections tend to focus on crop model performance under warming, with limited attempts to understand model performance solely in response to [eCO_2_]. For example^56^, evaluated the integrated effect of temperature and CO_2_ on wheat phenology and yield using CERES and N-Wheat. Similarly, interactive effect of CO_2_ and temperature on soybean [*Glycine max* (L.) Merr.].

water use efficiency (WUE), foliage temperature, canopy resistance and evapotranspiration were studied earlier^57^. Root Zone Water Quality Model (RZWQM2) was used to model current and future climate change effects on winter wheat production but again they studied CO_2_ fertilization and warming effects in combination^58^. However, a systematic comparison of [eCO_2_] responses, independent of temperature, of crop models often used for climate change projections has not been attempted to our knowledge. The present study was designed to evaluate the performance of five process-based crop models (APSIM-Wheat, CropSyst, DSSAT-CERES-Wheat, EPIC and STICS) under different levels of [eCO_2_]. The objectives of the present study were to (i) quantify impact of [eCO_2_] on winter wheat biomass and yield and (ii) bring/suggest accuracy in the models’ response to [eCO_2_] and determine whether a multi-model ensemble approach would minimize uncertainty in climate change simulation.

## Results

### Biomass response to eCO_2_

The simulated biomass results at the high rainfall site (Pullman) depicted bias among models in response to [eCO_2_]. All models provided different standard errors (Table 1). Significant difference among models for simulated biomass at ambient carbon dioxide concentration was observed with highest value simulated by CropSyst and lowest by DSSAT. However, with carbon dioxide concentration at 700 µmol mol^−1^ the simualted biomass started increasing with the largest response in STICS, followed by CropSyst and APSIM. A smaller response for biomass was simulated in EPIC followed by DSSAT at 700 µmol mol^−1^. Nevertheless, the overall response to [eCO_2_] was similar among models (Table 1). [eCO_2_] increased the simulated biomass on average by 34% among all models. The percentage increase of simulated biomass from ambient CO_2_ concentration (400 µmol mol^−1^) to 1000 ppm was 39, 37, 34, 33 and 25% for STICS, DSSAT, CropSyst, APSIM and EPIC, respectively.

**Table 1:** The simulated historical (1979–2010) mean performance of winter wheat biomass (kg ha^−1^) under ambient (aCO_2_) and elevated carbon dioxide (eCO_2_) at a high rainfall site near Pullman, WA, at a low rainfall site near Lind, WA and at an irrigated site near Moses Lake, WA.

	aCO_2_ (µmol mol^−1^)	eCO_2_ (µmol mol^−1^)					
	400	500	600	700	800	900	1000
**Pullman WA**							
APSIM	14501 (416)	16928 (462)	17975 (344)	18929 (514)	20422 (526)	21490 (535)	21538 (543)
CropSyst	16661 (538)	19060 (574)	20987 (585)	22609 (583)	23899 (578)	24825 (568)	25332 (563)
DSSAT	14081 (600)	15601 (661)	17149 (722)	18599 (779)	19935 (831)	21171 (878)	22239 (917)
EPIC	14863 (194)	16352 (204)	17502 (211)	18399 (216)	19101 (221)	19649 (224)	20074 (227)
STICS	16305 (380)	19241 (471)	21574 (529)	23370 (562)	24839 (596)	25954 (621)	26865 (643)
**Lind WA**							
APSIM	7230 (334)	9300 (341)	10119 (344)	11133 (369)	11725 (372)	12234 (378)	12627 (385)
CropSyst	7003 (403)	8173 (467)	9248 (519)	10285 (563)	11223 (599)	11971 (625)	12359 (644)
DSSAT	6395 (541)	7239 (601)	8112 (664)	8940 (723)	9703 (782)	10424 (836)	11057 (884)
EPIC	7622 (519)	8997 (565)	10348 (620)	11768 (682)	13016 (698)	14128 (707)	14629 (727)
STICS	5396 (318)	6515 (360)	7750 (486)	8814 (349)	9364 (424)	9801 (472)	10001 (457)
**Moses Lake, WA**							
APSIM	19301 (312)	20518 (328)	21338 (339)	21921 (344)	22347 (346)	22677 (349)	22947 (350)
CropSyst	23198 (421)	25109 (448)	26186 (462)	26854 (471)	27302 (477)	27623 (482)	27860 (485)
DSSAT	18216 (600)	20094 (586)	21911 (571)	23444 (569)	24693 (587)	25776 (595)	26615 (602)
EPIC	19135 (343)	21233 (398)	22105 (562)	22757 (500)	23596 (502)	24566 (544)	24127 (553)
STICS	16242 (218)	17552 (226)	18723 (234)	19755 (240)	20640 (245)	21432 (251)	22138 (258)

(Note: Standard errors (SE) of mean of the simulated data are in parentheses).

Biomass accumulation at the low rainfall site (Lind) was more responsive to [eCO_2_] compared to the high rainfall site among all models (Table 1). Overall, biomass accumulation among models was similar but a higher biomass was simulated in the EPIC crop model. Table 1 showed that at [aCO_2_], STICS responded lowest for biomass accumulation followed by DSSAT while on increasing [CO_2_] to 1000 ppm, biomass accumulation increased significantly. The standard error for biomass accumulation in response to [eCO_2_] remained highest in DSSAT followed by EPIC. The range of standard error for DSSAT was 541 to 884 while for EPIC it was 519 to 727. On average, all models simulated 44% increase in biomass. The highest biomass percentage change from [aCO_2_] (400 µmol mol^−1^) to to [eCO_2_] (1000 µmol mol^−1^) was shown by EPIC (48%) followed by STICS (46%). However, overall order of increase was EPIC > STICS > CropSyst > APSIM > DSSAT.

The effect of [eCO_2_] on biomass accumulation at the irrigated site (Moses Lake) revealed that it did not increase significantly with increased [CO_2_] compared to the other two sites. The highest response to [eCO_2_] was observed for CropSyst which ranged from 23198 to 27860 kg ha^−1^. APSIM simulation for biomass accumulation in response to [eCO_2_] ranged from 19301–22947 kg ha^−1^ with standard error of 312–350. Similarly, DSSAT simulated wheat crop biomass was 18216 kg ha^−1^ at [aCO_2_] (400 µmol mol^−1^) which increased to 26615 kg ha^−1^ at 1000 ppm, [CO_2_]. The standard error for simulated biomass was highest for DSSAT (Table 1). Genearlly, all five crop models agreed with the assumptions that elevated atmsospheric CO_2_ concentrations increases crop biomass but the effect was more prominent at the low rainfall site (Lind) compared to the well watered site (Moses Lake). The average percentage change in response to [eCO_2_] by all models at the irrigated site was 22%. The highest percentage change in biomass from ambient to [eCO_2_] concentration was observed in DSSAT (32%) followed by STICS (27%). However, the lowest change was simulated in APSIM. The overall order of increase in biomass was DSSAT > STICS > EPIC > CropSyst > APSIM.

**Figure 1:**
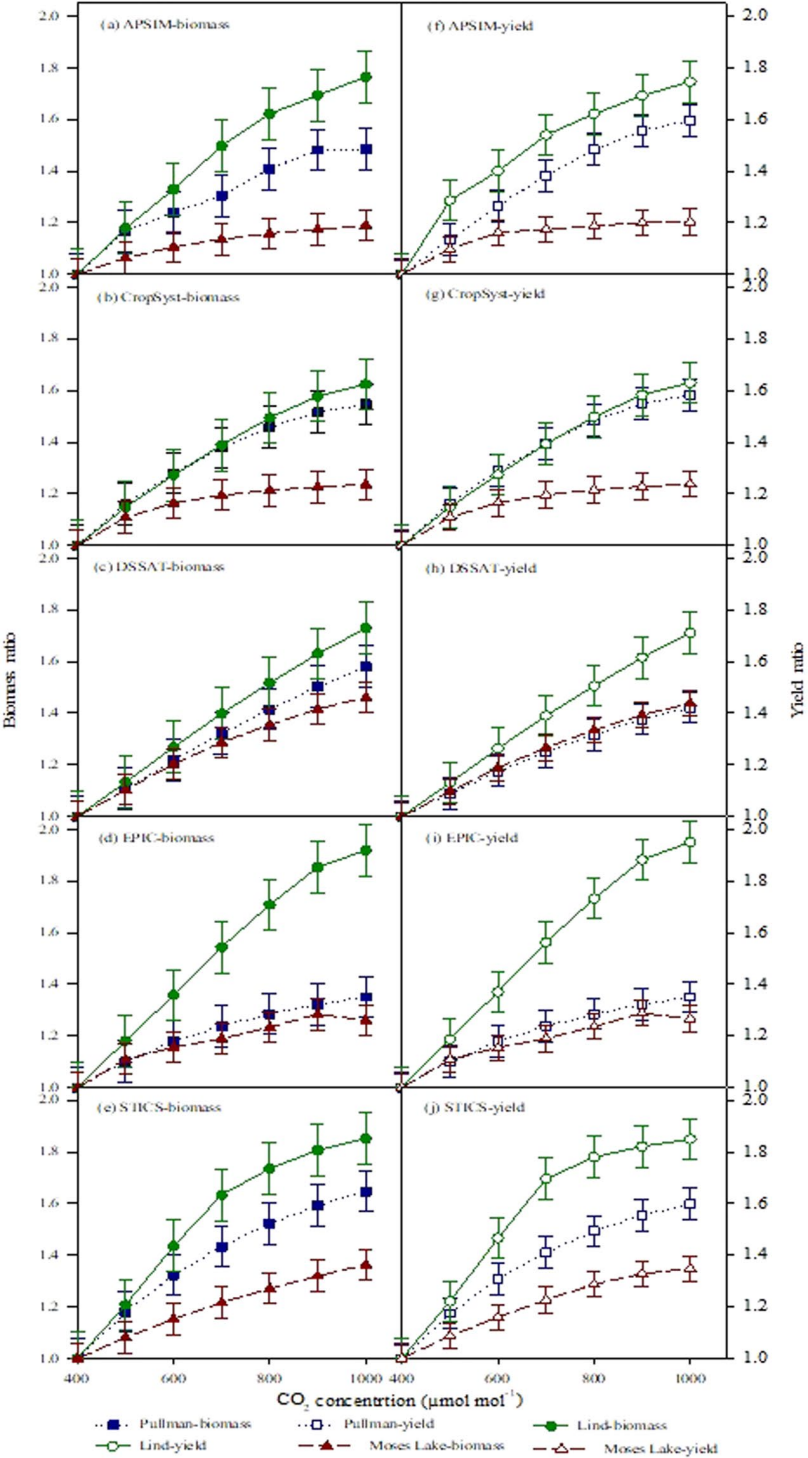
Biomass (**a**–**e**) and yield (**f**–**j**) ratios of wheat under elevated carbon-dioxide concentration [eCO_2_] for APSIM, CropSyst, DSSAT, EPIC, and STICS at three Pacific Northwest sites. Three coloured lines represents sites at Pacific Northwest with ± standard errors and each box represents Model outputs for Biomass and Yield. Means are averaged over three replicates.

The outcome of biomass ratio by APSIM against increased CO_2_ concentration showed an increasing trend with the highest response at the Lind compared to the irrigated one (Moses Lake). The biomass ratio remained similar between low and high rainfall sites at 500 µmol mol^−1^ but at higher concentrations it became significantly different (Fig. 1a). However, the trend was higher at the low rainfall site and lower at the high rainfall site after 900 µmol mol^−1^. The biomass ratio increase at the irrigated site remained close to 1.2 while at the low and high rainfall sites it reaches to 1.5 and 1.8, respectively (Fig. 1a).

The response of CropSyst simulation to elevated CO_2_ for biomass ratio revealed that it remained highest at the dryland site compared to the irrigated and high rainfall sites. The overall trend among all locations was increasing but at the irrigated site the increase was not too high (Fig. 1b). Overall, CropSyst and APSIM depicted similar trends for biomass ratio at all three locations.

**Figure 2:**
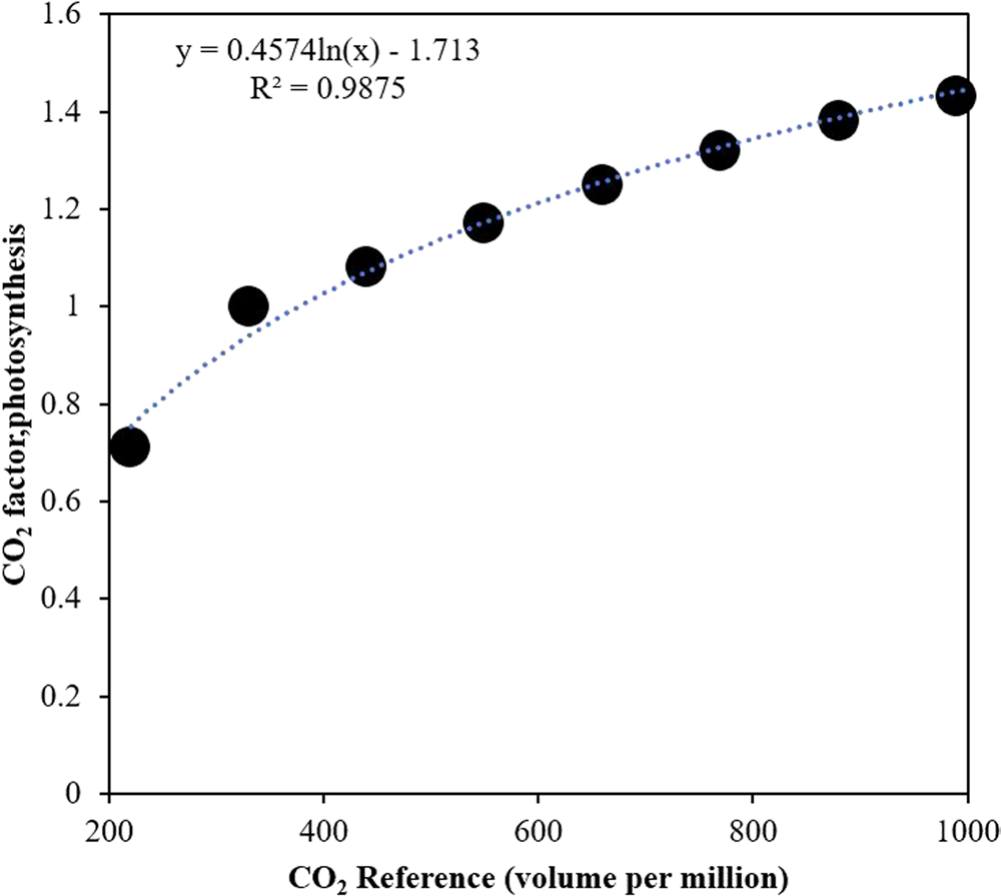
Asymptotic DSSAT response to elevated CO_2_. Wheat uses an asymptotic look-up multiplier on RUE for the relative response to elevated CO_2_ to produce biomass. The asymptotic look-up multiplier for modeled effects of elevated CO_2_ on RUE is given in the WHCER045.spe file. Regression equation was fitted to show the response of CO_2_ factor, photosynthesis with CO_2_ reference and validated by coefficient of determination (R^2^).

With the increased CO_2_ concentrations from baseline 400 µmol mol^−1^ to 1000 µmol mol^−1^, the DSSAT crop model depicted a linear increase in biomass ratio among all locations (Fig. 1c). However, like APSIM and CropSyst, the biomass ratio remained highest at dryland site followed by the high rainfall and irrigated sites. The difference among sites in response to [eCO_2_] was not too much as seen earlier in APSIM and CropSyst (Figs 1a and 2c). Similarly, in contrast to APSIM and CropSyst, the DSSAT response to [eCO_2_] increased linearly. To some extent, APSIM and CropSyst depicted sigmoid trends for simulated biomass ratio in response to [eCO_2_].

EPIC depicted an increasing trend for biomass ratio in response to [eCO_2_]. The trend was more prominent at the low rainfall site. The biomass ratio was similar at high rainfall and irrigated sites from 400 to 500 µmol mol^−1^ while at higher concentrations it increased at the high rainfall site compared to the irrigated site (Fig. 1d). However, the EPIC biomass ratio response for high rainfall and irrigated sites remained significantly different from the other models.

The STICS response to [eCO_2_] showed a similar increasing trend like other models for the dryland site followed by the high rainfall site (Fig. 1e). However, the response remained similar among high and low rainfall site up to 500 µmol mol^−1^ while at higher concentrations it increased sharply at the low rainfall site. Biomass ratio at the irrigated site increased linearly but remained lower than the other two sites.

### Yield response to eCO_2_

The impact of [eCO_2_] on grain yield showed increasing trends by all five crop models at the high rainfall site (Pullman). The highest yield at [aCO_2_] 400 µmol mol^−1^ was simulated by STICS (7101 kg ha^−1^) followed by APSIM (6368 kg ha^−1^). The lowest yield at [aCO_2_] was predicted by DSSAT (6214 kg ha^−1^). The range of standard errors at 400 µmol mol^−1^ was 209–239 with a highest standard error with EPIC. However, on moving from ambient CO_2_ concentration (400 µmol mol^−1^) to 700 µmol mol^−1^, the highest yield was simulated by STICS (10013 kg ha^−1^) followed by APSIM and CropSyst. The standard error range for grain yield at 700 µmol mol^−1^ was 255–323 with the highest standard error shown by STICS. The yield response at 1000 µmol mol^−1^ CO_2_ concentration showed that STICS simulated the highest yield (11353 kg ha^−1^) while the lowest was depicted by DSSAT (8835 kg ha^−1^). The standard error at 1000 µmol mol^−1^ CO_2_ ranged from 274–343 (Table 2). Overall, the average percentage increase in grain yield by all models from 400 to 1000 was 34%. However, the highest increase was observed in CropSyst and STICS (38%) followed by APSIM where it remained 37%. The lowest percentage change was observed for EPIC (26%) followed by DSSAT.

**Table 2:** The simulated historical (1979–2010) mean performance of winter wheat yield (kg ha^−1^) under ambient (aCO_2_) and elevated carbon dioxide (eCO_2_) at a high rainfall site near Pullman, at a low rainfall site near Lind and at an irrigated site near Moses Lake, WA.

	aCO_2_ (µmol mol^−1^)	eCO_2_ (µmol mol^−1^)					
	400	500	600	700	800	900	1000
**Pullman WA**							
APSIM	6368 (209)	7222 (235)	8040 (253)	8782 (264)	9417 (268)	9882 (271)	10132 (274)
CropSyst	6268 (225)	7223 (248)	8040 (264)	8782 (278)	9417 (289)	9882 (294)	10132 (294)
DSSAT	6214 (216)	6768 (232)	7298 (245)	7772 (255)	8184 (265)	8551 (272)	8835 (277)
EPIC	6148 (239)	6764 (243)	7239 (246)	7610 (248)	7900 (250)	8127 (251)	8302 (253)
STICS	7101 (226)	8340 (278)	9284 (303)	10013 (323)	10606 (345)	11044 (351)	11353 (343)
**Lind WA**							
APSIM	2339 (134)	2757 (136)	3112 (146)	3503 (158)	3795 (171)	3963 (183)	4129 (195)
CropSyst	2842 (167)	3320 (194)	3759 (217)	4189 (237)	4584 (253)	4897 (266)	5058 (275)
DSSAT	2643 (226)	2988 (251)	3339 (277)	3675 (304)	3980 (329)	4269 (354)	4524 (376)
EPIC	3159 (249)	3748 (264)	4329 (265)	4937 (267)	5472 (311)	5948 (351)	6162 (370)
STICS	2369 (150)	2892 (135)	3480 (236)	4017 (165)	4217 (206)	4314 (236)	4381 (267)
**Moses Lake, WA**							
APSIM	7853 (166)	8609 (168)	9109 (179)	9209 (185)	9311 (193)	9413 (197)	9429 (203)
CropSyst	10559 (184)	11424 (195)	11911 (202)	12213 (206)	12416 (208)	12561 (210)	12668 (212)
DSSAT	8322 (350)	9125 (352)	9888 (357)	10541 (367)	11107 (377)	11594 (387)	11970 (395)
EPIC	7995 (142)	8866 (164)	9224 (230)	9510 (293)	9877 (305)	10297 (345)	10120 (367)
STICS	7353 (155)	7989 (158)	8529 (161)	9020 (165)	9466 (168)	9768 (192)	9915 (227)

(Note: Standard errors (SE) of mean of the simulated data are in parentheses).

The simulated grain yield increased significantly as a function of CO_2_ concentration at the low rainfall site (Table 2). The average yield increase among all models from [eCO_2_] to 1000 µmol mol^−1^ was 45%, comparatively higher than high rainfall site where it was 34%. The highest percentage increase was shown by EPIC (49%) and STICS (46%) followed by CropSyst (44%), APSIM (43%) and DSSAT (42%). The grain yield at [aCO_2_] was in the range of 2339–3159 kg ha^−1^ with the highest yield simulated by EPIC. Similarly, standard error ranged from 134–249 with the highest error depicted by EPIC. On increasing CO_2_ concentration, yield increased linearly with the highest response shown by EPIC. The yield response trend at 700 µmol mol^−1^ CO_2_ revealed that EPIC (4937 kg ha^−1^) predicted highest wheat yield followed by CropSyst (4189 kg ha^−1^), STICS (4017 kg ha^−1^), DSSAT (3675 kg ha^−1^) and APSIM (3503 kg ha^−1^). The standard error at 700 µmol mol^−1^ CO_2_ concentration was in the range of 158–304 with highest standard error depicted by DSSAT. Similarly, at 1000 µmol mol^−1^ CO_2_ the yield remained highest (6162 kg ha^−1^) for EPIC followed by CropSyst (5058 kg ha^−1^). The standard error ranged from 195–376 with the highest error depicted by DSSAT.

The models’ simulated results of grain yield for the irrigated site (Moses Lake) in response to elevated [eCO_2_] showed a linear relationship (Table 2). The highest response was with CropSyst at ambient as well as eCO_2_ compared to other models. The highest yield at [aCO_2_] was 10559 kg ha^−1^ with a standard error of 184. However, the highest standard error (350) was observed for DSSAT with the grain yield of 8322 kg ha^−1^. Overall, the average grain yield at [aCO_2_] combind over all models was 8608 kg ha^−1^. The range of standard error at 400 µmol mol^−1^ CO_2_ was 142–350. On increasing CO_2_ from 400 to 700 µmol mol^−1^, the yield increased among all models. The maximum yield was simulated by CropSyst (12213 kg ha^−1^) followed by DSSAT at 700 µmol mol^−1^ CO_2_. The range of standard error at 700 µmol mol^−1^ CO_2_ was 165–367 with highest standard error for DSSAT. A similar trend was observed at 1000 µmol mol^−1^ CO_2_ with standard error in the range of 169–395. The average percentage increase in grain yield among all models from 400 to 1000 µmol mol^−1^ CO_2_ was 22% which was almost 50% less than the Lind. The highest percentage increase was with DSSAT (30%) followed by STICS (26%). However, the percentage change in yield from [aCO_2_] to [eCO_2_] concentration in EPIC was 12%. APSIM and CropSyt percentage response to [eCO_2_] in comparison to [aCO_2_] was similar (17%).

### Water use efficiency response to eCO_2_

The effects of elevated [CO_2_] on WUE depicted positive trend by all five crop models with the highest WUE at [eCO_2_] compared to [aCO_2_] at all sites. WUE results at Pullman showed that crop model STICS simulated highest WUE at all level of [CO_2_] as compared to all other models. However, the lowest WUE was shown by crop model EPIC in response to [eCO_2_] (Table 3). Water use efficiency results at low rainfall site revealed that APSIM simulated the lowest value of WUE while EPIC depicted the highest value at all level of CO_2_. However, under irrigated conditions maximum WUE was simulated by CropSyst while it remained minimum for crop model STICS and APSIM. Generally, all model showed increasing trends for WUE in relationship with an increased concentration of CO_2._ On average among models [eCO_2_] resulted to the increased WUE as when we move from [aCO_2_] to [eCO_2_] at all sites and it was 12–19% for Pullman, 11–20% for Lind and 14–18% for Moses Lake respectively (Table 3).

**Table 3:** Water use efficiency (WUE) of winter wheat (kg ha^−1^ mm^−1^) under ambient (aCO_2_) and elevated carbon dioxide (eCO_2_) at a high rainfall site near Pullman, at a low rainfall site near Lind and at an irrigated site near Moses Lake, WA.

	aCO_2_ (µmol mol^−1^)	eCO_2_ (µmol mol^−1^)					
	400	500	600	700	800	900	1000
**Pullman WA**							
APSIM	12.29 ± 0.31	13.94 ± 0.27	15.52 ± 0.31	16.95 ± 0.36	18.18 ± 0.37	19.07 ± 0.39	19.56 ± 0.40
CropSyst	12.01 ± 0.30	13.94 ± 0.27	15.52 ± 0.31	16.95 ± 0.35	18.18 ± 0.38	19.07 ± 0.40	19.56 ± 0.41
DSSAT	12.00 ± 0.32	13.06 ± 0.35	14.09 ± 0.39	15.00 ± 0.37	15.80 ± 0.38	16.51 ± 0.39	17.06 ± 0.41
EPIC	11.87 ± 0.29	13.06 ± 0.33	13.97 ± 0.37	14.69 ± 0.33	15.25 ± 0.36	15.68 ± 0.39	16.03 ± 0.42
STICS	13.71 ± 0.33	16.10 ± 0.40	17.92 ± 0.38	19.33 ± 0.35	20.47 ± 0.39	21.32 ± 0.39	21.92 ± 0.43
**Lind WA**							
APSIM	9.67 ± 0.35	11.39 ± 0.43	12.86 ± 0.43	14.48 ± 0.43	15.68 ± 0.45	16.38 ± 0.46	17.06 ± 0.47
CropSyst	11.74 ± 0.37	13.71 ± 0.42	15.53 ± 0.45	17.30 ± 0.48	18.94 ± 0.49	20.23 ± 0.51	20.90 ± 0.52
DSSAT	10.92 ± 0.33	12.35 ± 0.39	13.80 ± 0.41	15.19 ± 0.42	16.45 ± 0.43	17.64 ± 0.45	18.69 ± 0.47
EPIC	13.05 ± 0.36	15.49 ± 0.44	17.89 ± 0.47	20.40 ± 0.50	22.61 ± 0.52	24.58 ± 0.55	25.46 ± 0.59
STICS	9.79 ± 0.32	11.95 ± 0.43	14.38 ± 0.43	16.60 ± 0.45	17.43 ± 0.46	17.83 ± 0.43	18.10 ± 0.47
**Moses Lake, WA**							
APSIM	13.09 ± 0.35	14.35 ± 0.40	15.18 ± 0.41	15.35 ± 0.42	15.52 ± 0.43	15.69 ± 0.44	15.72 ± 0.45
CropSyst	17.60 ± 0.37	19.04 ± 0.41	19.85 ± 0.43	20.36 ± 0.44	20.69 ± 0.45	20.94 ± 0.47	21.11 ± 0.48
DSSAT	13.87 ± 0.35	15.21 ± 0.39	16.48 ± 0.41	17.57 ± 0.43	18.51 ± 0.44	19.32 ± 0.45	19.95 ± 0.43
EPIC	13.33 ± 0.36	14.77 ± 0.41	15.37 ± 0.43	15.85 ± 0.44	16.46 ± 0.45	17.16 ± 0.46	16.87 ± 0.48
STICS	12.26 ± 0.37	13.32 ± 0.42	14.22 ± 0.43	15.03 ± 0.45	15.78 ± 0.47	16.28 ± 0.42	16.56 ± 0.45

(Note: Standard errors (SE) of mean of the simulated data are represented as ±).

The yield ratio for APSIM at three sites showed that the value of this ratio was a function of CO_2_ (Fig. 1f). However, this response was significantly different among the several climatic locations. The highest yield ratio was obtained for water stress conditions where it increased from 1 to 1.8 followed by the high rainfall site (Pullman) where the change was from 1 to 1.6. However, the lowest yield ratio was obtained at the irrigated site where it remained in the range of 1–1.2.

The yield ratio response to [eCO_2_] for CropSyst depicted higher increasing trend under water stress conditions compared to irrigated (Fig. 1g). The yield ratio response between high and low rainfall was similar at 500 µmol mol^−1^ CO_2_ but at higher concentrations, the increase was more prominent at the low rainfall site. The yield increase ratio at the water stress site was from 1 to 1.8 while under high rainfall conditions it was from 1 to 1.5. The response to [CO_2_] at the irrigated site remained in the range of 1 to 1.2. The yield ratio response of APSIM was similar to CropSyst (Fig. 2d–f).

The simulated yield ratio response of DSSAT revealed that it was more linear and directly related to [CO_2_] under water stress conditions than APSIM and CropSyst (Fig. 1h). However, the increased ratio (1–1.7) was a little bit lower than DSSAT. The other difference in DSSAT simulated yield ratio response compared to APSIM and CropSyst was that it showed similar trends for high rainfall and irrigated conditions. The range was from 1–1.4.

The yield ratio trend simulated by EPIC was significantly different among locations (Fig. 1i). The highest yield ratio (1–2.0) was observed under water stress conditions. EPIC response to [eCO_2_] was similar to other crop models (APSIM, CropSyst and DSSAT) particularly under water stress conditions. However, under high rainfall and irrigated conditions, the yield ratio remained similar untill 500 µmol mol^−1^ above which it increased at the high rainfall site. The range of increase at the high rainfall site was 1–1.4. In contrast to other crop models, EPIC depicted a decreasing trend under irrigated conditions.

With the increase in CO_2_ from baseline 400 µmol mol^−1^ to 1000 µmol mol^−1^, the STICS model depicted yield increase as a function of CO_2_ concentration at all locations (Fig. 1j). The dominant trend was observed at the dryland site similar to previous model results followed by yield ratio outcomes at the high rainfall site. The lowest ratio was obtained under non-stressed conditions (Fig. 1j). The range of yield ratio increase for the dryland site was 1–1.8 while for high rainfall site it was 1–1.6. The increased yield ratio at the irrigated site was in the range of 1–1.3.

## Discussion

Crop models can be used to see the impact of various climatic variables on crop biomass and yield under changing climate. These models have been continuously refined to give a realistic picture of different climatic variables impacts on crop production. However, models have limitations in the prediction of crop responses to global change factors^59^. Challinor *et al*.^60^ and Soussana *et al*.^33^ emphasized the need to improve crop models for assessments of climate change. Since most of the models need improvements in climate impact assessment, we evaluated five different process-based models under eCO_2_. The models we tested simulated increased biomass on average by 34% but this trend was greatest under dryland conditions as compared to irrigated. This difference could be because of lower stomatal conductance and reduced transpiration which might result in higher water use efficiency. Earlier work concluded that [eCO_2_] alone will increase biomass and yield in C3 crops as photosynthesis of C3 plants are not CO_2_‐saturated and photosynthesis rates increases under [eCO_2_]. The two major plant responses e[CO_2_] are (i) increased net photosynthesis with a consequent increase in crop growth and yield, and (ii) decreased stomatal conductance and increase crop water use efficiency^12,61^. Similar to our work some studies suggest that these responses become very important under water-limited conditions and they reported greater e[CO_2_] response by plants under drier conditions because of greater water use efficiency^61^. Furthermore, buffering action of e[CO_2_] has been reported against heat waves which resulted in the increased crop production under semi‐arid environments. Specific genotypic adaptation strategies have been suggested to capture the positive effects of elevated [CO_2_] under drier conditions^62^. Thus, under dryland conditions, small amounts of water could contribute to enhanced photosynthate production and its translocation to the grain^63^. Manderscheid *et al*.^64^ reported increased biomass under [eCO_2_] by 17% with significant CO_2_ × N interaction and this can be increased further up to 30% by +200 ppm of CO_2_. Hence, similar to our modeling outcomes it has been suggested that under eCO_2_ crop water use of well-fertilized wheat will improve due to reduction in seasonal evapotranspiration. Fitzgerald *et al*.^65^ reported a large response to e[CO_2_] and suggested field level research to provide a detailed mechanistic understanding for adapting crops to climate change. O’Leary *et al*.^29^ reported that elevated CO_2_ (700 μmol mol^−1^) increased leaf area index (21%), photosynthetic area index (25%) and biomass (23%). However, our biomass response to eCO_2_ was slightly higher than reported by Ainsworth and Long^66^ and was consistent with Kimball^9^. e[CO_2_] experiments in Nanjing China who reported a 13.6% increase in biomass, but their maximum CO_2_ was 550 μmol mol^−1^. The relatively small increase in their experiment was because of a decrease in biomass between heading and maturity while positive effects were found during pre-anthesis growth phases which resulted in higher grain yield^67^. Carboxylation and oxidation of ribulose 1,5-bisphosphate (RuBP) are going on in C_3_ plants due to enzyme Rubisco. Under e[CO_2_] inhibition of Rubisco oxygenation/photorespiration occurs which results in higher photosynthesis and biomass production. According to Long *et al*.^68^ a 200 ppm rise in CO_2_ could theoretically result in a 40% increase in photosynthesis per unit of leaf area which is considerably higher than experimental results^69^. Fitzgerald *et al*.^70^ tested the hypothesis that biomass and yield response to e[CO_2_] is greater in semi‐arid agroecosystems or not and they measured biomass and yield increase up to 79% well above the measured highest response (34%) by Liu *et al*.^71^. Shimono *et al*.^72^ suggested different prescreening techniques which could be used for identification of elevated CO_2_-responsive genotypes. However, among these techniques, we suggest process-based crop models as a good option to test CO_2_-responsive genotypes among different plant species. Kumagai *et al*.^73^ proposed that the Finlay–Wilkinson relationship could be used as a pre-screening criterion for e[CO_2_] responsiveness, which is regression based, while crop models provide a detailed mechanistic approach.

Variable yield responses were simulated by models under e[CO_2_], however, the largest response was observed at Lind compared to Moses Lake, where it was lowest (Table 2). This magnitude of yield response was higher than the findings of O’Leary *et al*.^29^ and Yang *et al*.^74^ who reported an average increase of 26% and 24.8% respectively. Deryng *et al*.^75^ reported increased crop yield under rising CO_2_ which might be because of enhanced photosynthesis and reduced water use. Simulation modeling is a complex process as it involves interactions of multiple factors thus simulation by individual crop model might contain uncertainties. Quantifying the impact of climate change (e.g. elevated CO_2_, rising temperature and variability in rainfall) on crop yield by single crop model is problematic as suggested by different researchers^44,76,77^. Therefore, ensemble multi-models for simulation of climate change impacts could be a good option as proposed in the present study and in earlier work. Transpiration efficiency (TE) needs to be considered as an important mechanism in models for explaining response of increased crop yield under dryland conditions and elevated CO_2_^78^. Similar to our findings yield stimulation due to e[CO_2_] was reported by Fitzgerald *et al*.^70^ but it was in the higher range of 24% to 70% depending upon environment (Fig. 1). Similarly, they reported that heat wave effects could be ameliorated under e[CO_2_]. Long *et al*.^68^ reported that [CO_2_] of 200 ppm above ambient could result in a 10–20% increase in crop yield. However, Ziska *et al*.^79^ studied the impact of [CO_2_] on quantitative and qualitative traits of wheat varieties and concluded that elevated CO_2_ resulted in increased seed yield while grain and flour protein declined. Similarly, lower nutritional quality in grains of non-legume crops was reported by Jin *et al*.^80^ due to e[CO_2_]. C-N-P stoichiometry of terrestrial plants to the rising CO_2_ concentration showed that concentrations of N, P and N:P will decrease by −9.73%, −3.23% and −7.23% while C and C:N will increase by +2.19 and +13.29% respectively^81^.

Water use efficiency is the amount of grain produced per unit of water used by the crop. Elevated CO_2_ concentration improved the water use efficiency with more positive effect under stress condition compared to the well-watered plants. This could be due to the fact that under water-limiting and [eCO_2_] conditions photosynthetic CO_2_ uptake response increases resulting in the higher CO_2_ fixation. In present studies, the highest WUE was simulated by the crop model EPIC at low rainfall site while for all other models increasing trend was observed. Different studies^10,19,29,82^ discussed the positive effect of [eCO_2_] on WUE which could be due to reduced stomatal conductance resulting to lower canopy transpiration and crop water use^12,83,84^. However, negative WUE were reported by earlier researchers and they suggested upgradation in the code of models to have a sufficiently strong effect of CO_2_ on stomatal conductance and on transpiration^85^. Similarly, stomatal resistance also regulates photosynthesis and transpiration and it affected by CO_2_ and vapor pressure deficit (VPD)^86^. Thus, leaf level CO_2_ exchange rate and stomatal closure have association with VPD which increases with temperature and have a strong relationship with radiation use efficiency (RUE)^87–90^. Our study suggests integrating the effects of all these crucial factors in the models so that they can simulate results in a biologically realistic manner. Since most of the models are unable to accommodate these factors particularly under [eCO_2_] thus we recommend using model ensemble or adapt physiological mechanisms in the model. Therefore, it is necessary to consider these findings in process-based models to have better response under eCO_2_. The response to e[CO_2_] in our studies suggested further evaluation/improvement of models, particularly under stressed conditions. Models could be improved by local calibration with consideration of radiation use efficiency (RUE) and transpiration efficiency(TE) methods of biomass accumulation. Similarly, temperature and light intensity interactions with eCO_2_ should be considered, which will render models more effective for future climate change studies.

## Conclusion

Model response to [eCO_2_] showed significant increases in biomass and yield of wheat. Overall models were able to capture [eCO_2_] response but with differences in response to environmental conditions. The response was higher under dryland conditions compared to irrigated which could be because of lower stomatal conductance and transpiration resulting in higher water use efficiency. However, to have more accurate simulation results from models it is important to calibrate the model under local dryland conditions and consider the interactive effect of light intensity with [eCO_2_]. In the future models could be used to pre-screen large numbers of germplasms for [eCO_2_] responsiveness at relatively low cost. Process-based crop models have variability in the simulation of crop response to elevated CO_2_ with a greater difference under water-stressed conditions. An ensemble approach will increase the accuracy of model response to elevated CO_2_.

## Materials and Methods

Five process-based crop models were evaluated in the present study: APSIM-Wheat^91,92^, CropSyst^93,94^, DSSAT-CERES-Wheat^95^, EPIC^96^ and STICS^97,98^. These models were chosen because of their wide use in climate change studies and their ready availability. The focus of this evaluation was on biomass and yield responses to [eCO_2_], with attention to changes in crop transpiration, biomass and grain yield. The relevant details of the approaches used in these models to simulate the response to [eCO_2_] are described below.

### APSIM

APSIM (Agricultural Production system SIMulator) is a dynamic daily time step model that can simulate crop growth, development and yield using different management and biophysical modules. It is capable of simulating soil C, N, P and water dynamics in interactions with different management/crops systems driven by daily meteorological data (Solar radiation, maximum and minimum temperatures, rainfall). Radiation-use efficiency (RUE) approach is used to calculate daily potential production of crops which is then limited to actual above ground biomass production on a daily time step basis by N, P and soil water availability^92^. Soil water balance is simulated by cascading bucket approach of CERES^99^ through SOILWAT and SWIM3 modules^100^. APSIM wheat includes potential response to [eCO_2_] of RUE and transpiration efficiency (TE). The dynamic RUE response with varying [eCO_2_] is a non-linear relationship while TE is linearly related to [eCO_2_]. A multiplier (*plant_rue_co*2*_modifier*) increases RUE with [eCO_2_] as shown in Eq. 1, where [CO_2_ref] is the reference atmospheric CO_2_ concentration at which the multiplier’s value is one. An increase in [eCO_2_] from 350 to 700 ppm at a mean temperature of 20 °C will bring a 21% increase in RUE^101^. RUE is scaled by the ratio of light limited photosynthetic response at the elevated CO_2_ compared with CO_2_ at 350 ppm.

1 $$C3\_co2\_rue\_{modifier}=\frac{([eC{O}_{2}]-{\rm{\Gamma }})\,([{\rm{CO}}2{\rm{ref}}]+2\cdot {\rm{\Gamma }})}{([eC{O}_{2}]+2\cdot {\rm{\Gamma }})\,([{\rm{CO}}2{\rm{ref}}]-{\rm{\Gamma }})}$$

where [*eCO*_*2*_] is the elevated target CO_2_ concentration (ppm), [CO_2_ref] refers to the reference CO_2_ concentration and Γ is the CO_2_ compensation point with ${\rm{\Gamma }}=\frac{163-{T}_{av}}{5-0.1\cdot {T}_{av}}$ where *T*_*av*_ is the mean temperature (°C). TE increases by 37% with an increase in [eCO_2_] from 350 to 700 ppm. These values are based on glasshouse experiments with wheat^102^ supported and validated by FACE field experiments^103^ as reported by Reyenga *et al*.^104^.

### CropSyst

CropSyst is multi-year, multi-crop, daily time-step cropping system model. It can simulate the effect of management, soil and climate on crop growth, development and yield. Detail of CropSyst model is available on the website (http://modeling.bsyse.wsu.edu/CS_Suite_4/CropSyst/index.html). The [eCO_2_] effect on crop biomass can be simulated by CropSyst which relies on biomass accumulation under experimental elevated CO_2_ (C_a_). C_a_ in CropSyst is expressed as the ratio of growth under elevated C_a_ (C_eo_) to growth under control baseline C_a_ (C_bo_). The subscript, “o”, represents experimental conditions. A Michaelis-Menten-type equation models relative biomass growth (G_r_) in response to C_a_ (Eq. 2).

2 $${G}_{r}=\frac{{C}_{a}{G}_{x}}{{C}_{a}+s}$$

G_r_ (relative biomass growth similar to leaf photosynthesis response to intercellular CO_2_ concentration) will be less than 1.0 if C_a_ < C_bo_ and vice versa. G_x_ (maximum growth increases relative to baseline conditions) and s can be obtained after considering G_r_ = 1.0 (C_a_ = C_bo_) and equal to G_ro_ when C_a_ = C_eo_ (Eqs 3–4).

3 $${G}_{x}=\frac{{C}_{bo}+s}{{C}_{bo}}$$

4 $$s=\frac{{C}_{eo}{C}_{bo}({G}_{ro}-1)}{{C}_{eo}-{G}_{ro}{C}_{bo}}$$

The value of *e* for a given elevated C_a_ (e_e_) is calculated by considering e_e_ = eGr. Stomatal resistance (*u)* is also considered in CropSyst under elevated C_a_ as it reduced transpiration ratio *F* (elevated to baseline *Ca* crop transpiration per unit leaf area). Thus, *u* under elevated *Ca* (*ue)* is given by *ue* = *uGr*/*F*. Both *e* and *u* are parameters specified for baseline *Ca* conditions. Stockle *et al*.^86^, presented calculation of stomatal resistance as a function of C_a_. Meanwhile, F could be calculated as the ratio of elevated to baseline Ca considering aerodynamic and canopy resistance^94^.

### CERES-Wheat

CERES-Wheat embedded in Decision Support Systems for Agrotechnology Transfer (DSSAT, v 4.7) uses daily time step from planting to maturity to simulate the growth and development of crops^105^. Potential growth (G_p_) is a function of photosynthetically active solar radiation (PASr) and its interception (Si) by crops but limited by suboptimal temperature, soil water, N and P deficits. Cardinal temperature approach has been used in CERES-Wheat to simulate temperature effects on crop growth and grain filling with an optimum temperature of 34 °C^106,107^. CERES-Wheat uses an asymptotic look-up multiplier on RUE for the relative response to elevated CO_2_ to produce biomass. The asymptotic look-up multiplier for modeled effects of elevated CO_2_ on RUE is given in the WHCER045.spe file (Fig. 2). The CERES-Wheat model simulates the effect of elevated CO_2_ on actual transpiration by increasing stomatal resistance as a function of CO_2_ concentration.

An approach for reducing transpiration as a function of rising CO_2_ was developed for the DSSAT models in the early 1990s by J.W. Jones and L.H. Allen (personal communication, see TRANS routine of DSSAT code). The computations include equations for leaf stomatal resistance (*R*_s_) response to 330 ppm or current CO_2_, whole canopy stomatal resistance (*R*_c_) to reference or current CO_2_ (dividing *R*_s_ by total LAI), and canopy boundary layer resistance (*R*_a_) as a function of LAI. Finally, a ratio effect (*T*_ratio_) of CO_2_ (current CO_2_ versus 330 ppm reference CO_2_) to reduce daily transpiration is computed in the following equation, considering the psychrometric constant *(δ)*, gamma *(γ)*, canopy resistances (*R*_c_), and boundary resistance (*R*_a_) (Eq. 5).

5 $${{T}}_{{\rm{ratio}}}=(\delta +\gamma \times (1.0+{R}_{c}/{R}_{a}))/(\delta +\gamma \times (1.0+{R}_{c}//{R}_{a}))$$

### EPIC

Environmental Policy Integrated Climate (EPIC) model is a cropping systems model which can simulate crop growth, development and yield in response to climatic variables, crop and soil management. The phenological development of plant is function of temperature and it is based on daily heat unit accumulation. Potential growth is linked with interception of solar radiation and estimated by Beers law^96^. EPIC uses logistic equation to simulate the effect of [eCO_2_] on RUE (Eq. 6).

6 $$RUE=\frac{C{O}_{2}\cdot 100}{[C{O}_{2}+{b}_{1}\exp (\,-\,{b}_{2}C{O}_{2})]}$$

where RUE is radiation-use efficiency and CO_2_ is the [eCO_2_]. The parameters b_1_ and b_2_ can be calculated by solving the equation for two known points (RUE and CO_2_) on the response curve^86^.

### STICS

STICS (Simulateur mulTIdisciplinaire pour les Cultures Standard) is a generic soil-crop model that can simulate crop growth, soil water and N balance on a daily time step^108,109^. Crop growth is determined through plant carbon accumulation, solar radiation interception by the foliage and transformation into different plant parts. Growing degree days are used to simulate crop phenology and it is a function of temperature, water and N stress. The water budget calculates water in soil and crop and water stress indices reduces leaf growth and net photosynthesis of crop. Radiation use efficiency (RUE) concept is used to calculate shoot biomass while overall biomass accumulation is function of phenology, temperature, water and N stress. STICS uses a RUE approach to simulate the effect of [eCO_2_] on biomass production as proposed by Stӧckle *et al*.^86^. The effect of [eCO_2_] on stomatal resistance is applied on a model adapted from Shuttleworth and Wallace^110^. STICS directly calculates daily above ground biomass which is the net result of photosynthesis, respiration and root/shoot partitioning. This daily accumulation is a function of intercepted radiation according to a parabolic law involving maximum RUE^109^. Maximal RUE values are given as input parameters in STICS which depend on species and phenological stages. For example, RUE values are low during the juvenile phase, and RUE for oil seed crops diminishes during the filling phase.

### Study sites

This study was conducted for three variable climatic sites in US Pacific Northwest (PNW) using the above five crop models. The sites include Pullman, Moses Lake and Lind. The altitude of Pullman is 756 m at latitude 46°44′N and longitude of 117°10′W and it comes under high rainfall. The average historical (1979–2010) annual rainfall in Pullman is 474.70 mm with crop seasonal rainfall of 518 mm while actual evapotranspiration at pullman was 474.66 mm with maximum, minimum and average temperature of 12.51, 1.92 and 7.21 °C. Moses Lake is an irrigated site with altitude of 326 m and longitude 47°32′N and latitude of 119°54′W. The average historical temperature during the winter wheat crop season at Moses Lake was 8.68 °C with minimum and maximum tempearure of 3.03 and 14.32 °C, respectively. Seasonal rainfall received during historical simulation time period at Moses lake was 200 mm while 400 mm of irrigation was also applied during wheat growing season. Lind is a low rainfall site with average annual rainfall of 216.15 mm and seasonal rainfall of 242 mm. The altitude of Lind is 505.35 m at latitude 47°00′N and longitude of 118°56′W. Historical average temperature at Lind was 7.1 °C with minimum and maximum temperatures of 1.14 and 13.05 °C respectively. The historical actual evapotranspiration at Lind was 251.2 mm.

### Soil data

The soil of the Pullman site was silty clay loam with bulk density of 1.35 g cm^−3^. Soil texture at Lind was coarse silt loam with bulk density of 1.31 g cm^−3^. Field capacity in the specific root zone at Pullman was 0.30 mm mm^−1^ while wilting point water in specific root zone was 0.12 mm mm^−1^. The soil series of Pullman was Palouse which is a deep well drained soil. The textue of Lind was coarse silt loam having sand, silt and clay percentages of 21.7, 70.8 and 7.5, respectively. The bulk density at Lind was 1.31 g cm^−3^ with field capcity of 0.33 mm mm^−1^ and wilting point of 0.007 mm mm^−1^.The soil series at Lind was Lind which is a deep poorly drained soil. The irrigated site, Moses Lake, was in the Ephrata soil series, and had sandy loam soil texture with drain upper limit of 0.40 mm mm^−1^ while the lower limit was 0.27 mm mm^−1^. Bulk density of soil at Moses Lake was 1.41 g cm^−3^.

### Models calibration

APSIM v. 7.7, CropSyst v.4.19.06, DSSAT v. 4.7, EPIC v. 0810 and STICS v.8.4 were calibrated to observed data for crop phenology, LAI, biomass and yield. Biomass and yield were calculated at maturity of crop. The input data used to calibrate the models and set initial soil water conditions are presented in Table 4. After calibration all models were used to simulate winter wheat phenology, biomass and yield for all three sites for the baseline years 1979–2010.

**Table 4:** Input data of study sites for model calibration.

Parameters	High Rainfall	Low rainfall	Irrigated
	Pullman	Lind	Moses Lake
**Input Data**			
Planting date	15-Oct	1st Sept	1st Sept
Seed rate (kg ha^−1^)	110	60	106
Plant population (plants m^−2^)	200	150	200
Fertilizer rate (Kg ha^−1^)	160–200	110–150	150–250
Row to row distance (cm)	25–30	40 to 55	40 to 55
Emergence	22-Oct	8-Sep	8-Sep
Winter dormancy	December to March	December to March	December to March
Spring green up	March	March	March
End Vegetative	6-Jun	18-May	18-May
Flowering	11-Jun	23-May	23-May
Begin GF	4-Jul	10-Jun	10-Jun
Begin Senescence	9^th^ July	15-Jun	15-Jun
Maturity	3rd August	10^th^ July	10^th^ July
Harvesting	14-Aug	19^th^ July	19^th^ July
Leaf area index (LAI)	6	4	6
Kernels per spike	40–50	30–38	40–50
Spikes per m^2^	400–600	275–300	400–600
Thousand kernel weight	45–55 gm	38–40 gm	45–55 gm
Biomass (Kg ha^−1^)	15879	7729	20577
Grain Yield (Kg ha^−1^)	6779	2870	8916
**Soil depth**	**Initial soil water (mm mm** ^**−1**^ **)**		
0–0.1 m	0.13	0.05	0.05
0.1–0.2 m	0.13	0.11	0.13
0.2–0.3 m	0.17	0.11	0.15
0.3–0.4 m	0.17	0.12	0.15
0.4–0.5 m	0.2	0.12	0.15
0.5–0.7 m	0.19	0.12	0.15
0.7–1.0 m	0.2	0.13	0.16
1.0–1.5 m	0.2	0.14	0.16

### [eCO_2_]

After calibration, biomass and grain yield of winter wheat was simulated for [eCO_2_] of 400, 500, 600, 700, 800, 900 and 1000 µmol mol^−1^. Water use efficiency (WUE) in kg ha^−1^ mm^−1^ were calculated by using following equations:

7 $$WUE\,at\,Pullman\,and\,Lind=\frac{Grain\,Yield}{Seasonal\,Rainfall\,}$$

8 $$WUE\,at\,Moses\,Lake=\frac{Grain\,Yield}{Seasonal\,Rainfall+Irrigation\,}$$

### Statistical analysis

Simulation outcome for winter wheat biomass and grain yield in response to [eCO_2_] during 1979–2010 was used to calculate average. Standard error was calculated for biomass and yield. The average biomass and yield at 400 ppm were considered as baseline. The ratio in biomass and yield change were calculated for all other concentrations of CO_2_ using 400 ppm as baseline. The ratio was plotted against CO_2_ concentration to see models’ response to [eCO_2_] and uncertainty among the models. Average with standard error for WUE was calculated in response to [eCO_2_].
